# Effect of N-3 Polyunsaturated Fatty Acids on Lipid Composition in Familial Hypercholesterolemia: A Randomized Crossover Trial

**DOI:** 10.3390/biomedicines10081809

**Published:** 2022-07-27

**Authors:** Liv Nesse Hande, Christian Kjellmo, Kristin Pettersen, Stefan Ljunggren, Helen Karlsson, Karin Cederbrant, Maritha Marcusson-Ståhl, Anders Hovland, Knut Tore Lappegård

**Affiliations:** 1 Department of Medicine, Nordland Hospital, 8092 Bodø, Norway; kjellmo@gmail.com (C.K.); anders.w.hovland@gmail.com (A.H.); knut.tore.lappegard@gmail.com (K.T.L.); 2 Faculty of Health Sciences, UiT The Arctic University of Norway, 9037 Tromsø, Norway; 3 Research Laboratory, Nordland Hospital, 8092 Bodø, Norway; kristin.pettersen2@nlsh.no; 4 Division of Occupational and Environmental Medicine, Department of Health, Medicine and Caring Sciences, Linköping University, 581 85 Linköping, Sweden; stefan.ljunggren@regionostergotland.se (S.L.); helen.m.karlsson@liu.se (H.K.); 5 Division of Inflammation and Infection, Department of Biomedical and Clinical Sciences, Linköping University, 581 85 Linköping, Sweden; karin@cederbrant.com; 6 Swedish Toxicology Sciences Research Center, 152 57 Södertälje, Sweden; maritha@marcusson-stahl.com; 7 Department of Clinical Medicine, UiT The Arctic University of Norway, 9037 Tromsø, Norway

**Keywords:** familial hypercholesterolemia, omega-3 fatty acids, lipids, cardiovascular disease, randomized trial

## Abstract

Individuals with familial hypercholesterolemia (FH) have an increased risk of cardiovascular disease. Treatment is mainly low-density lipoprotein cholesterol (LDL-C) reduction. How omega-3 polyunsaturated fatty acids (n-3 PUFAs) supplements affect lipoproteins in FH subjects is unknown. We hypothesized that a high-dose n-3 PUFA supplement would reduce atherogenic lipoproteins and influence the high-density lipoprotein cholesterol (HDL-C) function. We performed a randomized, double-blinded crossover study with 34 genetically verified FH individuals (18–75 years, clinically stable, statin treatment > 12 months). Treatment was 4 g n-3 PUFAs (1840 mg eicosapentaenoic acid and 1520 mg docosahexaenoic acid daily) or four capsules of olive oil for three months in a crossover design with a washout period of three months. The defined outcomes were changes in triglycerides, lipoproteins, lipoprotein subfractions, apolipoproteins, and HDL-C function. After treatment with n-3 PUFAs, total cholesterol, LDL-C, and triglycerides were reduced compared to placebo (*p* ≤ 0.01 for all). Total HDL-C levels were unchanged, but the subfraction of large HDL-C was higher (*p* ≤ 0.0001) after n-3 PUFAs than after placebo, and intermediate HDL-C and small HDL-C were reduced after n-3 PUFAs compared to placebo (*p* = 0.02 and *p* ≤ 0.001, respectively). No changes were found in apolipoproteins and HDL-C function. N-3 PUFAs supplements reduced atherogenic lipoproteins in FH subjects, leaving HDL-C function unaffected.

## 1. Introduction

Familial hypercholesterolemia (FH) is the most common monogenic disorder in the world [1]. Low-density lipoprotein cholesterol (LDL-C) reduction is the main treatment goal to prevent harmful long-term effects of LDL-C overload. However, only 50% of FH individuals reach their LDL-C treatment goal. Thus, many FH individuals live with a high residual risk of cardiovascular disease (CVD) [2]. All FH individuals are either in the high or very high cardiovascular risk category, and additional therapies to lower LDL-C in individuals with FH are needed [3].

The role of omega-3 polyunsaturated fatty acids (n-3 PUFAs) in cardiovascular health has been studied for decades. Early clinical trials indicated a reduction in CVD after n-3 PUFAs supplementation [4,5]. After the introduction of statins, the CVD-reducing effect of n-3 PUFAs has been debated [6,7]. The search for the most potent dosage and preparation of n-3 PUFAs is ongoing. The Reduction of Cardiovascular Events with Icosapent Ethyl—Intervention Trial (REDUCE-IT) showed an effect of icosapent ethyl supplement on cardiovascular outcomes [8]. In contrast, the Long-Term Outcomes Study to Assess Statin Residual Risk with Epanova in High Cardiovascular Risk Patients with Hypertriglyceridemia (STRENGTH) used a carboxylic acid preparation of eicosapentaenoic acid (EPA) and docosahexaenoic acid (DHA) and found no difference in major adverse cardiovascular events [9]. The effect of n-3 PUFAs on LDL-C is debated, ranging from potentially harmful to neutral in larger populations [10]. The triglyceride-lowering characteristics of n-3 PUFAs are less controversial, but the role of triglyceride-lowering in cardiovascular risk reduction has not been established [3,8,9,11]. The effect of n-3 PUFAs on lipoproteins is unclear for the FH population, as the trials available are limited and report divergent results [12,13,14]. 

It has been proposed that advanced lipoprotein testing can improve cardiovascular risk prediction, identify residual risk in high-risk patients, and guide lipid-lowering therapy. Available advanced lipoprotein testing ranges from subfractionating LDL-C and high-density lipoprotein cholesterol (HDL-C) particles and quantifying the particle number of LDL-C/HDL-C to a wide array of tests of HDL-C functionality. 

In this study, we investigated the effects of n-3 PUFAs on LDL-C and HDL-C subfractions, paraoxonase-1 (PON1) arylesterase activity, serum amyloid A1 (SAA1) levels, and cholesterol efflux capacity (CEC) in a group of patients with genetically verified FH. We hypothesized that a high-dose n-3 PUFAs supplement in this population would reduce LDL-C and triglycerides, reduce small, dense LDL-C particles (sdLDL-C), and improve HDL-C level and function compared to placebo. 

## 2. Materials and Methods

### 2.1. Trial Design and Interventions

The trial design and eligibility criteria have been previously reported [15]. In brief, the trial was randomized, double-blinded, and placebo-controlled and had a crossover design of nine months duration. Two treatment periods (three months each) were separated by a three-month washout period to minimize the potential carry-over effect. The inclusion criteria were age between 18 and 75 years, genetically verified FH, clinically stable disease, and statin treatment for at least 12 months. Exclusion criteria were noncompliance, pregnancy or fertility treatment, breastfeeding, cancer, and/or severe illness. Randomization was done at inclusion, with an allocation ratio of 1:1. We had a well-known study population with stable disease and expected a low drop-out rate; thus, a crossover design was chosen. The participants were patients at the lipid clinic at Nordland hospital (Bodø, Norway). An invitation letter was sent to the participants from the lipid outpatient clinic. Two research nurses collected blood samples and performed clinical tests. Three alternating physicians performed the physical examination.

The n-3 PUFAs and placebo were administered in the same manner in the two treatment periods; four capsules a day. The n-3 PUFAs capsule contained 460 mg EPA and 380 mg DHA (a daily dose of 1840 mg EPA and 1520 mg DHA). The placebo capsule contained olive oil. Both the n-3 PUFAs and the placebo were provided by BASF (Lysaker, Norway). The study medication was administered to the participants when they started each treatment period, and unused medicines were returned accordingly.

The primary outcome of this trial, as previously reported, was change in reactive hyperemia index assessed by peripheral arterial tonometry [15]. The outcome presented in this paper was a change in triglycerides, lipoproteins, and lipoprotein composition and function (predefined secondary outcomes).

The random allocation sequence of participants and the labeling of the study medication were provided by Apotekproduksjon AS (Oslo, Norway). To conceal the allocation sequence, the study medication was delivered in numbered containers. The project manager and a physician enrolled the participants in the trial. The study participants and care providers were blinded to the series of interventions. Apotekproduksjon AS kept the randomization key upon completion of the trial. A completed CONSORT checklist is available (Figure S1).

### 2.2. Blood Samples

Fasting blood samples were obtained by venipuncture (vacutainer tubes) at baseline, after the first treatment period, after washout, and after the second treatment period. Serum tubes were centrifugated at 2000× *g* for 10 min. The citrate vacutainers had 3.2% sodium citrate and were centrifugated at 3000× *g* for 20 min at 4 °C.

### 2.3. Lipoprotein Measurements

Serum levels of triglycerides and total, LDL, and HDL cholesterol were analyzed on an ADVIA 1800 system (Siemens Medical Solutions Diagnostics, Tokyo, Japan). The procedure was performed according to the manufacturer. Apolipoprotein A1 (ApoA1) and apolipoprotein B (ApoB) were measured by the ADVIA1800 system (Siemens Healthcare Diagnostics, Deerfield, IL, USA).

### 2.4. Lipoprotein Subfractions

**Figure 1:**
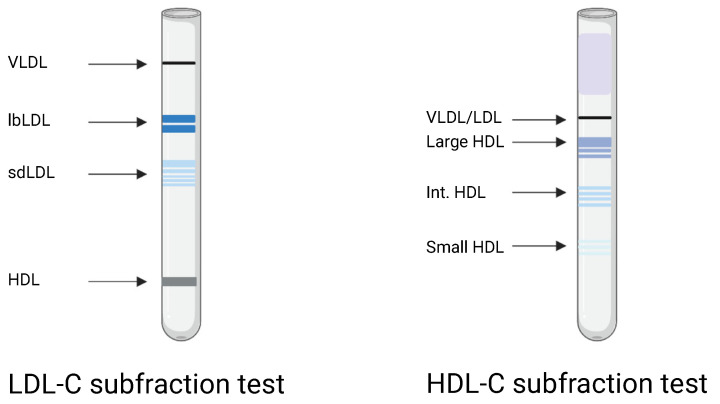
Graphical image of the Lipoprint LDL-C and HDL-C subfraction electrophoresis. VLDL: very low density lipoprotein. lbLDL: large, buoyant low-density lipoprotein cholesterol (LDL-C). sdLDL: small, dense LDL-C. HDL: high-density lipoprotein cholesterol. Int. HDL: intermediate HDL-C. Created with BioRender.com (accessed on 5 July 2022).

LDL-C and HDL-C subfractions were estimated by serum electrophoresis using the Lipoprint system (Lipoprint LDL system and Lipoprint HDL system, Quantimetrix Corporation, Redondo Beach, CA, USA). The analyses were performed according to the manufacturer’s instructions. The Lipoprint system provides lipoprotein subfractions divided into LDL-1 to LDL-7 and HDL-1 to HDL-10. LDL-1 and LDL-2 were classified as large, buoyant LDL-C (lbLDL-C) and LDL-3 to LDL-7 as small, dense LDL-C (sdLDL-C). HDL-1 to HDL-3 were categorized as large HDL-C, HDL-4 to HDL-7 as intermediate HDL-C, and HDL 8–10 as small HDL-C, as in Figure 1.

### 2.5. HDL-C Function

PON1 arylesterase activity was assessed in citrate plasma. Plasma was diluted at 1:80 using a salt buffer (20 mM Tris-HCl and 1.0 mM CaCl_2_ with pH 8.0). Twenty microliters of diluted plasma and 200 μL of phenylacetate solution (3.26 mM phenylacetate in salt buffer) were added to each well in a UV-transparent 96-well plate. The absorbance of produced phenol was measured at 270 nm in a FLUOstar plate reader (BMG Labtech, Ortenburg, Germany). The activity (U/mL) was calculated from the initial linear reaction, and an extinction coefficient of phenol of 1310 M-1 cm1 was used. 

The plasma SAA1 levels were measured by an enzyme-linked immunosorbent assay (DY3019-05, R&D Systems, Minneapolis, MN, USA) according to the manufacturer’s instructions. The absorbance was measured at 450 nm using a FLUOstar plate reader (BMG Labtech, Ortenburg, Germany). The cholesterol efflux capacity (CEC) was quantified with a MAK192 assay kit from Sigma-Aldrich (Saint-Louis, MO, USA), as previously described [16]. 

### 2.6. Statistical Analysis

Statistical work was performed using Prism version 8.4.3 (GraphPad Software Inc., La Jolla, CA, USA). Before the trial registration, a sample size calculation based on the primary outcome was performed. The period effect was tested by a two-sample *t*-test or Mann–Whitney test comparing the differences between the treatments in the two sequence order groups. Treatment–period interaction was evaluated by a *t*-test or a Mann–Whitney test comparing the average response in each sequence order group. The baseline values in the treatment sequence groups are presented as mean and standard deviation if normally distributed or as median and first and third quartile if not normally distributed. The normality in differences between treatment periods was assessed by the Shapiro–Wilk normality test. The values after n-3 PUFAs treatment and after placebo were compared by a paired *t*-test or Wilcoxon matched-pairs signed-rank test when appropriate. Confidence intervals (95%) were computed when the differences were symmetrically distributed. Correction for multiple comparisons was not performed. A 2-tailed *p*-level < 0.05 was considered significant.

## 3. Results

**Figure 2:**
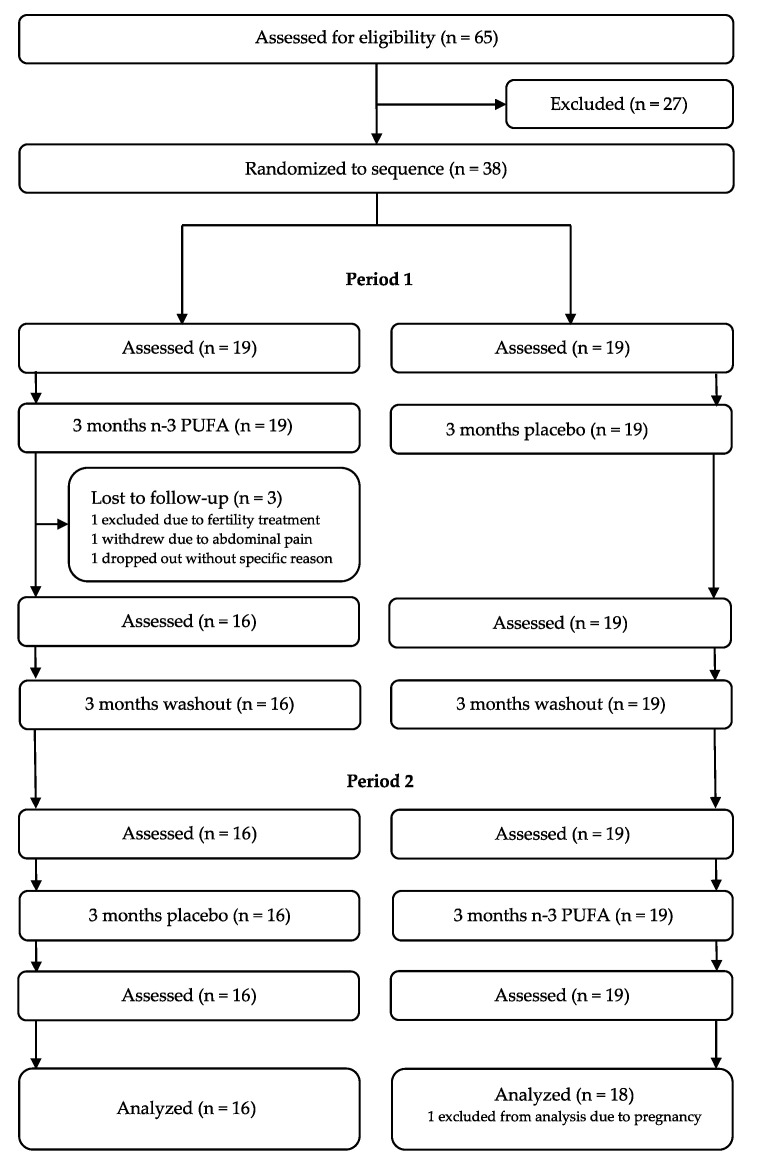
Participant flow diagram. N-3 PUFAs: omega-3 polyunsaturated fatty acids.

Of 65 subjects assessed for eligibility, 38 individuals were randomized to the sequence. The trial was conducted from September 2012 to July 2016. Three subjects left the trial, and one person was excluded from statistical analysis due to pregnancy, as shown in the participant flow diagram (Figure 2). Thirty-four participants (17 females and 17 males) with a mean age of 46.6 years completed the trial. The trial inclusion ended when the prespecified sample size (16 in each group) was reached. Sixteen started with n-3 PUFAs, and 18 started with placebo. Population characteristics and lipid changes from baseline have been previously published [15]. No important harms were detected.

### 3.1. Total Cholesterol, Triglycerides, and Lipoproteins

**Figure 3:**
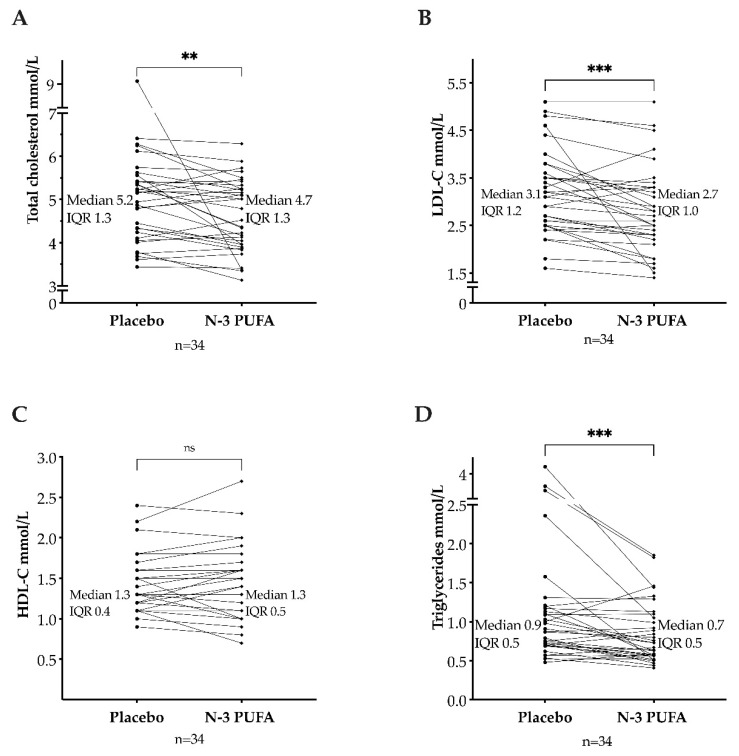
Total cholesterol (**A**), low-density lipoprotein cholesterol (LDL-C) (**B**), high-density lipoprotein cholesterol (HDL-C) (**C**), and triglycerides (**D**) after omega-3 polyunsaturated fatty acids (n-3 PUFAs) and placebo. IQR: interquartile range. ns = *p* > 0.05. ** = *p* ≤ 0.01. *** = *p* ≤ 0.001.

The values in Table 1 were obtained from the electrophoresis strip analysis by densitometric scanning using the Lipoware software. As shown in Table 1, the lipoproteins levels in the two sequence groups were comparable at baseline for treatment one and post-washout before the second treatment. The baseline distribution of study participants by intervals of LDL-C and triglycerides is presented in Table 2. Lipoprotein levels were compared after n-3 PUFAs treatment and after placebo. In median, the total cholesterol level was lower after n-3 PUFAs (median: 4.7 mmol/L; interquartile range (IQR): 4.0–5.3) than after placebo (median: 5.2 mmol/L; IQR: 4.2–5.3). This reduction was statistically significant (*p* = 0.006, median of differences: −0.24 mmol/L, Figure 3A). The LDL-C levels decreased after n-3 PUFAs (median: 2.7 mmol/L; IQR: 2.3–3.3) compared to placebo (median: 3.1 mmol/L; IQR: 2.5–3.7) (*p* < 0.001, with a median of differences of −0.2 mmol/L, 95% CI [−0.4, −0.1], Figure 3B). No difference in HDL-C levels was found between n-3 PUFAs treatment (median: 1.3 mmol/L; IQR: 1.1–1.6) and placebo (median: 1.3 mmol/L; IQR: 1.1–1.5) (*p* = 0.71, median of differences 0, with a 95% CI [−0.1, 0.1], Figure 3C). Triglycerides were lower after treatment with n-3 PUFAs (median: 0.7 mmol/L; IQR: 0.6–1.1) than after placebo (median: 0.9 mmol/L; IQR: 0.7–1.2) (*p* < 0.001, the median of differences −0.14 mmol/L, Figure 3D).

### 3.2. LDL and HDL Cholesterol Subfractions

**Figure 4:**
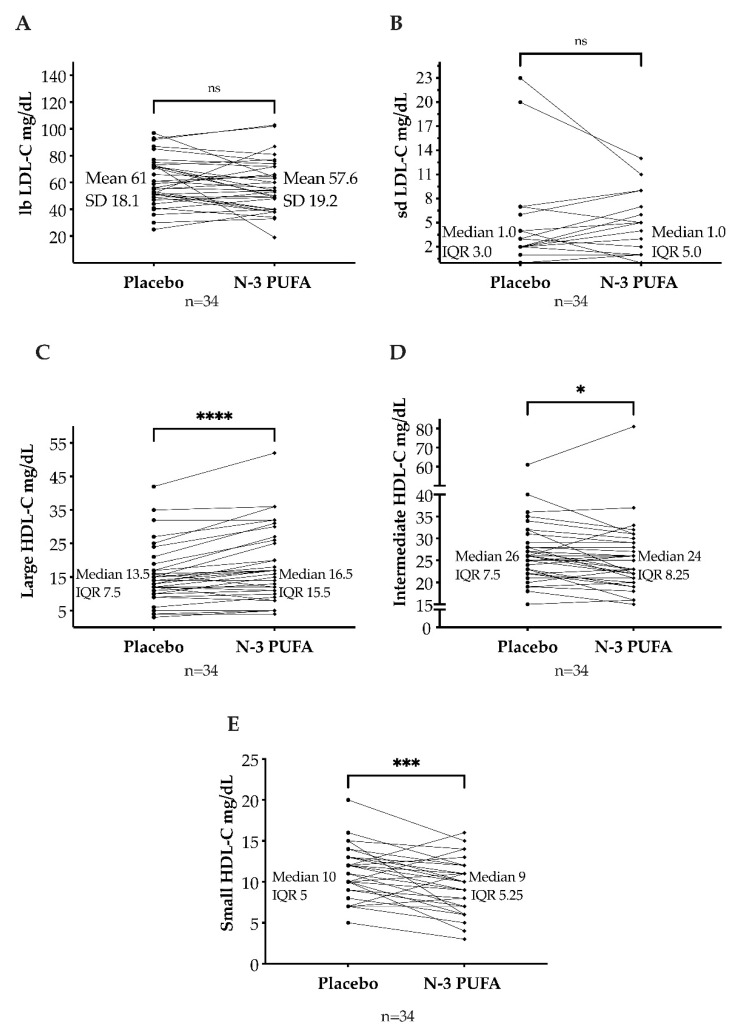
Large, buoyant low-density lipoprotein cholesterol (lbLDL-C) (**A**) and small, dense low-density lipoprotein cholesterol (sdLDL-C) (**B**) after omega-3 polyunsaturated fatty acids (n-3 PUFAs) and placebo. Large high-density lipoprotein cholesterol (large HDL-C) (**C**), intermediate HDL-C (**D**), and small HDL-C (**E**) after n-3 PUFAs and placebo. SD: standard deviation. IQR: interquartile range. ns = *p* > 0.05; * = *p* ≤ 0.05; *** = *p* ≤ 0.001; **** = *p* ≤ 0.0001.

The large, buoyant LDL-C subfraction was lower after n-3 PUFAs (mean: 57.5 mg/dL; SD: 19.2 mg/dL) than after placebo (mean: 61 mg/dL; SD: 18.1 mg/dL), but not statistically significant (*p* = 0.22, mean of differences = −3.4 mg/dL, 95% CI [−8.8; 2.1]). The small, dense LDL-C subfraction was unaffected by n-3 PUFAs (median: 1 mg/dL; IQR: 0–5) compared to placebo (median: 1 mg/dL; IQR: 0–3) (*p* = 0.33, with median of differences of 0. Figure 4A,B). 

In median, the large HDL-C subfraction was higher after n-3 PUFAs (median: 16.5 mg/dL; IQR: 10.8–26.3) than after placebo (median: 13.5 mg/dL; IQR: 10–17.5). This increase was statistically significant (*p* < 0.001, median of differences = 2.0 mg/dL with a 95% CI [1.0, 4.0]). The intermediate HDL-C subfraction was lower after n-3 PUFAs (median: 24 mg/dL; IQR: 20.8–29) than after placebo (median: 26 mg/dL; IQR: 22–29.5) (*p* = 0.02, median of differences = −1 mg/dL). Moreover, the small HDL-C subfraction decreased after n-3 PUFAs treatment (median: 9 mg/dL; IQR: 6–11.3) compared to placebo (median: 10 mg/dL; IQR: 8–13) (*p* < 0.001, median of differences = −2 mg/dL, Figure 4C–E).

### 3.3. Apolipoproteins and HDL-C Function

The parameters for apolipoproteins and HDL function at baseline before treatment one and post-washout are presented in Table 3. The N-3 PUFAs supplement did not affect the levels of ApoA1 and ApoB when compared to the placebo; Table 4. No difference in SAA1, PON1, and CEC was found after the n-3 PUFAs supplement compared to placebo; Table 4. 

## 4. Discussion

In this study, we found that n-3 PUFAs supplements in FH individuals reduced total cholesterol, LDL-C, and triglycerides. After the n-3 PUFAs supplement, the proportions of HDL-C subfractions changed. However, as assessed by SAA1, PON1, and CEC, the HDL-C function was unchanged during the trial.

Treatment with n-3 PUFAs reduced the LDL-C and TG levels in our FH population, followed by decreased total cholesterol. The LDL-C-lowering effect from n-3 PUFAs found in our trial differs from the results in previous trials investigating n-3 PUFAs supplementation in heterozygous FH individuals [12,13,14]. Two FH trials with four and eight weeks of combined EPA and DHA supplementation (5.1 g and 4 g) found no effect on LDL-C levels [12,14]. In contrast, six weeks of 1.2 g DHA supplement increased the LDL-C levels in children with FH and familial combined hyperlipidemia [13]. Short intervention periods, low sample sizes, use of DHA only, and lack of placebo comparison are possible explanations for the disparate results. In clinical trials, increased LDL-C levels after n-3 PUFAs supplements have been a concern. There are indications that the increase in LDL-C is related to DHA supplementation and not treatment with EPA [17]. This increase in LDL-C can reflect an increase in particle size rather than an increase in LDL-C concentration [18]. However, we found a reduction in LDL-C but no effect on particle size as large, buoyant LDL-C and small, dense LDL-C proportions were unchanged. 

N-3 PUFAs supplements are known for their triglyceride-reducing capacity. Increased triglycerides are not a hallmark of FH, and only 15% of our study population had triglycerides ≥1.7 mmol/L (150 mg/dL) at baseline. Hypertriglyceridemia is associated with increased atherosclerotic cardiovascular disease (ASCVD) risk [19], and triglycerides can be considered a marker for triglyceride-rich lipoproteins. Although epidemiological and genetic evidence supports a causal role for triglyceride-rich lipoproteins in the ASCVD pathway [11], evidence from clinical studies with triglyceride-reducing drugs is lacking. The triglyceride levels attained after treatment with an n-3 PUFAs supplement in the REDUCE-IT and the STRENGTH were comparable. However, only the REDUCE-IT showed a reduction in primary composite endpoint (CVD death, nonfatal myocardial infarction, nonfatal stroke, coronary revascularization, or unstable angina) after n-3 PUFAs treatment [8,9]. Therefore, it is suggested that the favorable effect found in the REDUCE-IT was not from a triglyceride-lowering pathway alone. N-3 PUFAs supplements are not recommended routinely in FH in the current European guidelines [3]. 

Small, dense LDL-C particles (sdLDL-C) are the advanced lipoprotein metric that has received the most attention over the last decades. Several large population-based studies found an association between elevated sdLDL-C and increased risk of CVD [20]. A higher atherogenic potential from small, dense LDL-C particles could be explained by an increased circulation time due to impaired interaction with the LDL-receptor, an increased susceptibility to undergo atherogenic modification (i.e., oxidization), and a greater propensity for transport into the arterial wall. Despite decades of research, the clinical significance of measuring sdLDL-C or other LDL-C subfractions is unknown, including among FH patients. Randomized diet trials of n-3 PUFAs in healthy volunteers have shown a reduction in sdLDL-C and an increase in LDL size [21,22]. In the present study, the concentration of sdLDL-C in the included patients was low at baseline, and we found no effect of n-3 PUFAs on either the small or large LDL-C subfractions.

Epidemiological and clinical trials are discordant regarding the prognostic value of measuring HDL subfractions. In different studies, both the smaller and the larger HDL-C subfractions have been proposed to be superior to HDL-C in CVD risk prediction. However, these studies have not been conducted in patients with FH. In the present study, we observed a significant change in the composition of HDL-C particles with a decrease in the smaller HDL-C particles and an increase in the larger HDL-C particles. The reverse transport of cholesterol (RCT) from peripheral tissue to the liver is probably the most critical mechanism for the protective effect of HDL-C on the development of CVD [23,24]. An early step in RCT is the efflux of cholesterol from macrophages to HDL particles [25]. Cholesterol efflux capacity is inversely correlated with hard vascular endpoints [26]. Versmissen et al. found that FH patients without CVD had higher CEC compared with non-FH siblings [27]. Ogura et al. found that CEC was independently and inversely associated with ASCVD in patients with heterogenous FH. The authors suggested that CEC could be a therapeutic target for preventing CVD in FH patients [28]. The smallest HDL particles have been proposed to be the most efficient mediators of cholesterol efflux [29,30]. Our data did not support this, as we observed significant changes in the large and small HDL subfractions without any changes in the cholesterol efflux capacity from n-3 PUFAs.

PON1 is an HDL-associated protein that has been proposed to have a protective effect on the development of CVD by reducing oxidative stress [31,32]. Prospective studies have shown that reduced PON1 activity is an independent risk factor for CVD [33,34]. PON1 activity is decreased in patients with FH compared to healthy controls [35]. We did not observe any differences in PON1 activity from n-3 PUFAs compared to placebo. 

SAA1 is an acute-phase protein that has been suggested to impair the anti-inflammatory properties of HDL-C, possibly by replacing its protective proteins [36]. SAA1 levels are elevated in patients with FH [37]. We did not observe any differences in serum SAA1 concentrations after n-3 PUFAs compared to placebo. 

The strengths of this trial are the crossover design, the well-known study population (single-center), and the treatment lengths (three-months treatment and at least a three-months washout). Several limitations need consideration. First, these are secondary endpoints and should be interpreted with care. Second, our study population was a combination of individuals in a primary and secondary prevention setting, placing our FH subjects in the high or very high cardiovascular risk category. Due to the sample size, we could not differentiate between the effect of n-3 PUFAs in the high-risk and the very high risk category. Third, the median LDL-C level at baseline was higher than recommended in the current European Guidelines for the management of dyslipidemia [3]. Thus, it is possible that the LDL-C-reducing effect of the n-3 PUFAs supplement would be nuanced in an FH population with lower LDL-C. However, we believe the LDL-C levels in our study population are representative of a clinical setting.

## 5. Conclusions

In this study of FH individuals, we found that n-3 PUFAs supplements reduced LDL-C and triglycerides. N-3 PUFAs did not change the LDL-C subfractions, but the large HDL-C subfractions increased, and the small HDL subfractions decreased. Despite changes in the HDL-C composition, we did not detect any alteration in the HDL function after the n-3 PUFAs supplement. N-3 PUFAs treatment has the potential to reduce LDL levels and change the HDL composition in FH subjects. The clinical benefit from these lipoprotein modifications remains to be elucidated, and further research is needed. 

## Figures and Tables

**Table 1:** Lipoproteins at baseline presented by sequence, treatment period, and total.

	Baseline					
	Treatment Sequence			Treatment Sequence		
Variable	N-3 PUFAs First, Then Placebo *n* = 16	Placebo First, Then N-3 PUFAs *n* = 18	Total *n* = 34	N-3 PUFAs First, Then Placebo *n* = 16	Placebo First, Then N-3 PUFAs *n* = 18	Total *n* = 34
	Before Treatment 1 (Month 0)			Before Treatment 2 (Month 6)		
**TC mmol/L**	4.8 ± 1.0	4.8 (4.4, 5.4)	4.7 (4.4, 5.4)	5.0 ± 1.2	5.0 ± 1.1	5.0 ± 1.2
**LDL-C mmol/L**	3.0 (2.7, 3.3)	3.1 (2.9, 3.5)	3.0 (2.8, 3.3)	3.2 ± 0.9	3.1 (2.6, 3.6)	3.2 (2.5, 3.7)
**HDL-C mmol/L**	1.3 (1.1, 1.5)	1.2 (1.0, 1.5)	1.2 (1.1, 1.5)	1.3 (1.1, 1.6)	1.2 (1.0, 1.6)	1.2 (1.1, 1.6)
**TG mmol/L**	0.7 (0.6, 1.3)	1.0 (0.8, 1.6)	0.8 (0.7, 1.4)	0.8 (0.6, 1.4)	0.9 (0.8, 1.3)	0.9 (0.7, 1.4)
**LbLDL-C mmol/L**	1.45 (1.2, 1.7)	1.45 (1.2, 1.6)	1.45 (1.2, 1.7)	1.55 ± 0.6	1.58 (1.0, 1.8)	1.58 (1.1, 1.9)
**LbLDL-C mg/dL**	56 (47, 67)	56 (46, 63)	56 (47, 65)	60 ± 22.3	61 (39, 68)	61 (41, 74)
**SdLDL-C mmol/L**	0.04 (0, 0.1)	0.05 (0.002, 0.1)	0.05 (0, 0.1)	0.03 (0, 0.1)	0 (0, 0.1)	0.01 (0, 0.1)
**SdLDL-C mg/dL**	1.5 (0, 3.0)	2.0 (0.8, 2.3)	2.0 (0, 3.0)	1.0 (0, 3.8)	0 (0, 4)	0.5 (0, 4.0)
**Large HDL-C mmol/L**	0.38 (0.2, 0.5)	0.34 (0.2, 0.5)	0.34 (0.2, 0.5)	0.44 ± 0.2	0.28 (0.2, 0.5)	0.3 (0.2, 0.6)
**Large HDL-C mg/dL**	14.5 (9.3, 20.5)	13 (6.5, 18.8)	13 (8.0, 19.5)	17.1 ± 8.8	11 (6.5, 17.8)	12 (9, 22)
**Int. HDL-C mmol/L**	0.68 ± 0.1	0.65 ± 0.2	0.66 ± 0.2	0.63 (0.6, 0.8)	0.65 ± 0.2	0.62 (0.5, 0.8)
**Int. HDL-C mg/dL**	26.3 ± 5.1	25.1 ± 7.9	25.7 ± 6.6	24.5 (22, 30)	25.1 ± 7.2	24.0 (21, 30)
**Small HDL-C mmol/L**	0.25 ± 0.1	0.3 ± 0.1	0.28 ± 0.1	0.25 ± 0.1	0.28 ± 0.1	0.27 ± 0.1
**Small HDL-C mg/dL**	9.7 ± 2.9	11.6 ± 3.2	10.7 ± 3.2	9.8 ± 4.2	10.9 ± 2.5	10.4 ± 3.4
**ApoA1 (µg/L)**	1.52 (1.3, 1.9)	1.49 (1.3, 1.8)	1.50 (1.3, 1.8)	1.48 (1.3, 1.8)	1.52 (1.2, 1.8	1.5 (1.3, 1.8)
**ApoB (µg/L)**	0.94 (0.8, 1.1)	1.0 (0.9, 1.1)	0.99 (0.8, 1.1)	0.95 (0.7, 1.2)	1.01 (0.9, 1.2)	1.0 (0.8, 1.2)
**ApoB/ApoA1**	0.61 (0.5, 0.7)	0.65 (0.5, 0.9)	0.62 (0.5, 0.8)	0.62 (0.5, 0.8)	0.64 (0.5, 0.9)	0.6 (0.5, 0.9)

Values presented are mean ± standard deviation or median (first quartile, third quartile) by normal or non-normal distribution. N-3 PUFAs: omega-3 polyunsaturated fatty acids. TC: total cholesterol. LDL-C: low-density lipoprotein cholesterol. HDL-C: high-density lipoprotein cholesterol. TG: triglycerides. LbLDL-C: large, buoyant low-density lipoprotein cholesterol. SdLDL-C: small, dense low-density lipoprotein cholesterol. Int. HDL-C: intermediate high-density lipoprotein cholesterol. From mg/dL to mmol/L, conversion factor 0.02586 was used. ApoA1: apolipoprotein A1. ApoB: apolipoprotein B.

**Table 2:** Baseline characteristics and intervals of LDL-C and triglycerides.

	Total	Group Starting with N-3 PUFAs	Group Starting with Placebo
**Number of patients, *n* (*n* female)**	34 (17)	16 (7)	18 (10)
**LDL-C < 1.8 mmol/L**	1 (0)	0	1 (0)
**LDL-C 1.8 to <2.6**	5 (2)	3(1)	2(1)
**LDL-C 2.6 to <3.0**	7 (5)	5 (3)	2 (2)
**LDL-C 3.0 to <4.9**	19 (9)	7 (3)	12 (6)
**LDL-C ≥ 4.9**	2 (1)	1 (0)	1(1)
**Triglycerides < 1.7 mmol/L**	29 (14)	15 (6)	14 (8)
**Triglycerides ≥ 1.7 mmol/L**	5 (3)	1(1)	4 (2)
**ApoB/ApoA1 > 0.9**	4 (2)	1 (0)	3 (2)
**Established ASCVD**	9 (4)	4 (1)	5 (3)

N-3 PUFAs: omega-3 polyunsaturated fatty acids. LDL-C: low-density lipoprotein cholesterol. Established ASCVD: established atherosclerotic cardiovascular disease defined as previous acute coronary syndrome (myocardial infarction or unstable angina), stable angina, coronary revascularization, other arterial revascularization procedures, stroke and transitory ischemic attack, and peripheral arterial disease.

**Table 3:** High-density lipoprotein function at baseline presented by sequence, treatment period, and total.

	Baseline					
	Treatment Sequence			Treatment Sequence		
Variable	N-3 PUFAs First, Then Placebo *n* = 16	Placebo First, Then N-3 PUFAs *n* = 18	Total *n* = 34	N-3 PUFAs First, Then Placebo *n* = 16	Placebo First, Then N-3 PUFAs *n* = 18	Total *n* = 34
	Before Treatment 1 (Month 0)			Before Treatment 2 (Month 6)		
**SAA1 (µg/mL)**	0.99 (0.5, 2.4)	2.2 (0.9, 4.1)	1.4 (0.7, 3.1)	1.1 (0.9, 1.8)	2.4 (1.4, 3.7)	1.7 (0.9, 2.5)
**PON1 (U/mL)**	111 ± 33.2	109.9 ± 28.8	110.4 ± 30.5	115.7 ± 29.2	108.7 ± 23.3	112.0 ± 26.1
**SAA1/PON1**	0.01 (0.005, 0.02)	0.02 (0.01, 0.04)	0.01 (0.01, 0.03)	0.01 (0.01, 0.02)	0.02 (0.01, 0.04)	0.02 (0.01, 0.03)
**CEC (%)**	37.5 ± 4.2	38.6 ± 5.0	39.1 ± 4.6	38.1 ± 4.0	39.5 ± 3.9	38.9 ± 4.0

Values shown are median (interquartile range) or mean ± standard deviation. N-3 PUFAs: omega-3 polyunsaturated fatty acids. ApoA1: apolipoprotein A1. ApoB: apolipoprotein B. SAA1: serum amyloid A1. PON1: serum paraoxonase-1. CEC: cholesterol efflux capacity.

**Table 4:** Apolipoproteins and high-density lipoprotein function presented by treatment.

	N-3 PUFAs *n* = 34	Placebo *n* = 34	Treatment Difference (N-3 PUFAs−Placebo) with 95% Confidence Interval	*p*
**ApoA1 (µg/L)**	1.51 (1.3, 1.7)	1.55 (1.3, 1.9)	−0.07 [−0.12 to 0.05]	0.29
**ApoB (µg/L)**	0.91 (0.8, 1.2)	1.03 (0.8, 1.2)	−0.06 [−0.13 to 0.01]	0.09
**ApoB/ApoA1**	0.57 (0.5, 0.7)	0.58 (0.5, 0.8)	−0.009 [−0.04 to 0.04]	0.91
**SAA1 (µg/mL)**	1.36 (1.0, 2.6)	1.68 (1.0, 2.7)	−0.11 [−0.59 to 0.11]	0.15
**PON1 (U/mL)**	107 ± 25.5	113 ± 31.0	−5.9 [−12.9 to 1.1]	0.10
**SAA1/PON1**	0.01 (0.01, 0.02)	0.02 (0.01, 0.2)	−0.002 [−0.006 to 0.003]	0.38
**CEC (%)**	38.5 ± 3.2	38.9 ± 3.5	−0.41 [−1.8 to 1.0]	0.57

N-3 PUFAs; omega-3 polyunsaturated fatty acids. Values are presented as median (interquartile range) or mean ± standard deviation. ApoA1: apolipoprotein A1. ApoB: apolipoprotein B. SAA1: serum amyloid A1. PON1: serum paraoxonase-1. CEC: cholesterol efflux capacity.

## Data Availability

The data presented in this study are available on request from the corresponding author.

## Supplementary Materials

The following supporting information can be downloaded at: https://www.mdpi.com/article/10.3390/biomedicines10081809/s1, Figure S1: CONSORT 2010 checklist of information to include when reporting a randomised trial.
